# SGLT2 inhibitors in CKD: are they really effective in all patients?

**DOI:** 10.1093/ndt/gfaf051

**Published:** 2025-03-07

**Authors:** Paola Romagnani

**Affiliations:** Nephrology and Dialysis Unit, Meyer Children's Hospital IRCCS, Florence, Italy; Department of Biomedical, Experimental and Clinical Sciences “Mario Serio”, University of Florence, Florence, Italy

**Keywords:** CKD, diabetic kidney disease, glomerulonephritis, obesity, proteinuria

## Abstract

Sodium-glucose cotransporter 2 (SGLT2) inhibitors effectively slow chronic kidney disease (CKD) progression and reduce cardiovascular events. However, their efficacy across all CKD subgroups remains uncertain. Major clinical trials primarily included overweight or obese patients with advanced CKD, where sodium retention, volume expansion and glomerular hyperfiltration are key disease drivers. In contrast, many underrepresented CKD subgroups, such as Alport syndrome or most immune-mediated glomerular disorders, often affect lean individuals whose CKD progression is not linked to these mechanisms. Emerging evidence suggests that the renal benefits of SGLT2 inhibitors may depend on body mass index (BMI), with greater effects observed in patients with higher BMI, while those with BMI <25 may show minimal/no benefit. This raises concerns about their applicability in lean, non-diabetic CKD patients, whose disease progression may involve alternative pathways, such as inflammation, autoimmunity or genetic abnormalities. Animal studies further suggest that SGLT2 inhibitors provide limited renal protection in certain genetic and immune-mediated kidney diseases. Additionally, molecular data indicate that SGLT2 expression is predominantly restricted to the proximal tubule, implying a limited role in CKD driven by non-hyperfiltration mechanisms. While SGLT2 inhibitors have revolutionized CKD treatment in diabetes, obesity and heart failure, their role in lean, non-diabetic patients remains unclear. Dedicated clinical trials are needed to assess their efficacy in underrepresented CKD subgroups, including pediatric patients, and ensure evidence-based, personalized treatment strategies.

A meta-analysis of numerous large clinical trials has provided robust evidence that Sodium-glucose cotransporter 2 (SGLT2) inhibitors significantly delay the progression of chronic kidney disease (CKD) and reduce cardiovascular events in patients with CKD, regardless of diabetes status [1]. These findings have driven the regulatory approval of SGLT2 inhibitors with broad indications for adult CKD, not including CKD patients with polycystic kidney disease. However, the universal applicability of these benefits remains uncertain. Do these benefits extend uniformly to all CKD patients, or are there other subpopulations where efficacy is limited or uncertain?

## ROBUST EVIDENCE FROM TRIALS BUT LIMITED REPRESENTATION

The Dapagliflozin and Prevention of Adverse Outcomes in Chronic Kidney Disease (DAPA-CKD) and Empagliflozin in Patients With Chronic Kidney Disease (EMPA-KIDNEY) trials form the cornerstone of SGLT2 inhibitor approvals [2, 3]. In DAPA-CKD, 32.4% of participants were non-diabetic [2], while EMPA-KIDNEY included 54% [3]. Among non-diabetic participants in EMPA-KIDNEY, the hazard ratio for kidney disease progression or cardiovascular death was 0.82 (confidence interval 0.68–0.99), closely paralleling the 0.64 (0.54–0.77) observed in diabetic participants [3]. These results have significantly expanded the therapeutic scope of SGLT2 inhibitors beyond diabetes.

Despite these compelling results, both trials predominantly included overweight or obese patients with advanced CKD. In DAPA-CKD, the mean body mass index (BMI) was 29.4 and glomerular filtration rate (GFR) 43.2 mL/min [2], while in EMPA-KIDNEY, the mean BMI was 29.7 and GFR 37.4 mL/min [3]. These populations shared common drivers of CKD progression, including glomerular hyperfiltration caused by sodium retention and expanded fluid volume (volemia)—mechanisms frequently associated with obesity [4], diabetes [5], heart failure [6] and advanced CKD [7] (Box 1).

Box 1. **Expanded volemia (fluid overload) and sodium retention are common but not universal pathophysiological features in CKD.**

**Obesity:** obesity is associated with increased activation of the renin–angiotensin–aldosterone system (RAAS), leading to sodium retention and volume expansion. This process results in systemic and intraglomerular hypertension, promoting renal fibrosis and contributing to CKD progression.

**Diabetes:** in diabetes, particularly type 2, hyperglycemia induces sodium retention through upregulation of SGLT2 in the renal proximal tubules. This upregulation increases sodium and glucose reabsorption, leading to expanded volemia and hypertension, which exacerbate renal damage.

**Heart failure:** heart failure often results in fluid overload due to the heart's reduced ability to pump blood effectively. This inefficiency activates compensatory mechanisms, including RAAS and sympathetic nervous system stimulation, leading to sodium retention and expanded volemia. These changes contribute to congestion and edema commonly observed in heart failure patients.

**Advanced CKD:** as CKD progresses, the kidneys’ ability to excrete sodium diminishes, resulting in sodium retention and volume overload. This accumulation leads to hypertension and edema, further impairing renal function and accelerating disease progression.

SGLT2 inhibitors were initially developed for diabetes treatment due to their dual hypoglycemic and diuretic effects [8]. By blocking glucose and sodium reabsorption in the proximal tubule, these drugs promote natriuresis, fluid loss and reduced glomerular hyperfiltration [8]. A recent study demonstrated that empagliflozin significantly reduced fluid overload in CKD patients, as measured by bioimpedance analysis, without affecting fat mass [9]. These findings underscore the central role of sodium retention and hyperfiltration in the observed benefits of SGLT2 inhibitors in these trials.

## GAPS IN EVIDENCE FOR UNDERREPRESENTED CKD SUBGROUPS

While hyperfiltration is a common mechanism of CKD progression, it does not explain all cases. Many CKD patients progress through alternative mechanisms, such as inflammation, immune dysregulation, genetic abnormalities or toxic exposures, or even by an hyperfiltration that causes stress on the podocyte favoring its detachment but does not associate with fluid retention [10–12]. These pathways are particularly relevant for conditions like glomerulonephritis, genetic kidney diseases, autoimmune disorders and congenital anomalies of the kidney and urinary tract (CAKUT).

**Figure 1:**
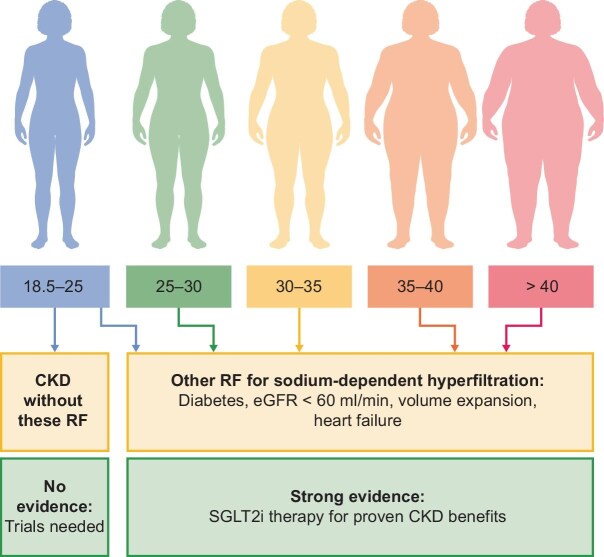
Patients with risk factors for CKD progression who benefit from gliflozin treatment and those who still need to be investigated.

Notably, conditions like Alport syndrome, immunoglobulin A (IgA) nephropathy and other immune-mediated glomerular diseases predominantly affect lean individuals in both Asia and Europe [13–17], contrasting sharply with the overweight, advanced CKD populations studied in DAPA-CKD and EMPA-KIDNEY. These underrepresented groups, often in earlier CKD stages, may progress through mechanisms unrelated to sodium retention and volume expansion (Fig. 1), requiring targeted therapies outside of the SGLT2 inhibitor spectrum.

## SUBGROUP ANALYSES AND POST-HOC FINDINGS HIGHLIGHT GAPS

Subgroup analyses of existing trials have suggested benefits of SGLT2 inhibitors for patients with BMI <25; however, these findings are confounded by significant biases [17, 18]. For instance, in the DAPA-CKD trial, 55% of participants were diabetic, and the mean estimated GFR (eGFR) was 42 mL/min [2, 17]. Similarly, in EMPA-KIDNEY, 46% of participants had diabetes, and 79% had an eGFR below 45 mL/min, indicating that most patients in these trials had conditions such as diabetes, obesity or advanced CKD—settings where SGLT2 inhibitors are already known to be effective [3, 18]. Post-hoc analyses of specific CKD subtypes reinforce these limitations. In the focal segmental glomerulosclerosis subgroup of DAPA-CKD, the average BMI was 30.7, and the mean eGFR was 40 mL/min [19], while in the IgA nephropathy subgroup, 17.5% of dapagliflozin-treated patients were diabetic, with a mean BMI of 27 and an eGFR of 43.8 mL/min [20]. Most trial participants with IgA nephropathy had obesity, diabetes or advanced CKD, contrasting sharply with the leaner cohorts of many trials on glomerulonephritis or Alport syndrome [13–17], which included patients with different risk factors for CKD progression. Similarly, in EMPA-KIDNEY, participants with glomerular diseases had a mean BMI of 27.2 and an eGFR of 42.4 mL/min [21]. These characteristics raise questions about the applicability of findings to lean, non-diabetic patients with early-stage CKD.

The Canagliflozin Cardiovascular Assessment Study (CANVAS) and Canagliflozin and Renal Events in Diabetes with Established Nephropathy Clinical Evaluation (CREDENCE) trials reported benefits for patients with BMI below 25; however, all participants in these studies were diabetic, further limiting their relevance to non-diabetic populations [22]. In contrast, the DIAMOND trial, which specifically evaluated dapagliflozin in non-diabetic CKD patients, found no significant effect on proteinuria in a cohort with a mean BMI of 28 and eGFR of 58.2 mL/min [23]. Notably, a study investigating SGLT2 inhibitors in patients with glomerulonephritis observed a significant reduction in proteinuria, but the effect was more pronounced in individuals with higher BMI, suggesting that the antiproteinuric efficacy of SGLT2 inhibitors may be enhanced in overweight and obese patients, likely due to the fluid retention often seen in this population [24]. Similarly, heart failure with reduced ejection fraction trials also demonstrated renal benefits from SGLT2 inhibitors in non-diabetic CKD patients [25, 26]. However, these studies focused on individuals with ejection fractions below 40%—a population marked by sodium retention and fluid overload. These findings are less likely to extend to CKD patients without hyperfiltration-related comorbidities, such as younger, leaner individuals with early-stage disease.

## BMI AS A KEY DETERMINANT OF SGLT2 INHIBITOR EFFICACY?

A recent nationwide epidemiological study investigated the kidney-protective effects of SGLT2 inhibitors compared with DPP4 inhibitors in over 6000 patients with type 2 diabetes [27]. While SGLT2 inhibitors were associated with a slower annual decline in eGFR, their protective effects were significantly dependent on BMI. Specifically, the study revealed a strong interaction between BMI and treatment efficacy, showing that the renal benefits of SGLT2 inhibitors were markedly greater in individuals with higher BMI, whereas the effect was negligible in those with BMI <25 [27]. This trend persisted even when alternative outcome measures, such as a 30% or 40% eGFR decline, were analyzed [27]. Moreover, this interaction effect was particularly evident in individuals with preserved kidney function, aligning with previous observations that SGLT2 inhibitors exert greater benefits in settings of sodium retention and fluid overload [27]. These findings raise critical questions about the generalizability of SGLT2 inhibitor benefits to lean, non-diabetic CKD patients. Given that many glomerular diseases primarily affect leaner individuals [13–17], the limited effect of SGLT2 inhibitors in patients with normal BMI calls for a reassessment of their role in this population.

## INSIGHTS FROM MOLECULAR AND ANIMAL STUDIES

Molecular and animal studies provide further context for the limitations of SGLT2 inhibitors in CKD subtypes where hyperfiltration is not the dominant driver of progression. Expression of SGLT2 has been reported in a limited number of cell types beyond the proximal tubule, such as cardiomyocytes [28], hepatocytes [29], peritoneal cells [30] and even podocytes [31]. However, single-cell RNA sequencing datasets reveal that SGLT2 mRNA expression is predominantly restricted to the kidney, even in diabetes (https://humphreyslab.com/SingleCell/displaycharts.php), suggesting the amount of SGLT2 expressed by these cells even in inflammatory conditions is minimal in comparison to that expressed by the proximal tubule. Protein data from the Human Protein Atlas corroborate these findings, showing minimal SGLT2 expression in tissues outside the proximal tubule (https://www.proteinatlas.org/ENSG00000140675-SLC5A2/tissue). This restricted expression suggests that effects beyond the kidney are unlikely to play a significant role in the renoprotective benefits of SGLT2 inhibitors.

Animal models reinforce these findings. In mouse models of Alport nephropathy, SGLT2 inhibitors showed no additive benefit when combined with RAS blockers and were less effective than RAS blockers alone [31, 32]. Similarly, in APOL1 nephropathy models, SGLT2 inhibitors had no significant impact on CKD progression [31]. Finally, renoprotective mechanisms-of-action of SGLT2 inhibition in diabetic kidney disease do not apply to chronic oxalosis, a non-diabetic form of CKD [33]. Together, these findings highlight the mechanistic constraints of SGLT2 inhibitors and their limited applicability to CKD subtypes driven by alternative pathophysiologies.

## BRIDGING THE KNOWLEDGE GAPS IN CKD MANAGEMENT

The current evidence strongly supports the use of SGLT2 inhibitors for CKD patients with hyperfiltration-related conditions, including diabetes, obesity, advanced CKD or heart failure. Notably, further analyses from EMPA-KIDNEY suggest that these drugs are effective even in non-proteinuric patients, though their benefits are more pronounced in those with proteinuria [34]. Hyperfiltration can occur without proteinuria, particularly in early diabetes or obesity, which aligns with the characteristics of EMPA-KIDNEY's cohort.

However, CKD patients with alternative disease mechanisms, such as lean, non-diabetic individuals, particularly if with early-stage CKD, represent a subgroup where the efficacy of SGLT2 inhibitors remains uncertain. Post-hoc subgroup analyses and animal studies highlight the need for a more nuanced understanding of the drugs’ mechanisms in underrepresented populations.

Moreover, it is essential to promote clinical trials in other underrepresented groups, such as patients on hemodialysis or peritoneal dialysis and kidney transplant recipients, as well as in patients that were not at all included in these trials, like polycystic kidney diseases. These populations face unique challenges and mechanisms of CKD progression that are unlikely to align entirely with those observed in current trials. Without dedicated studies, extending the use of SGLT2 inhibitors to these groups remains speculative and unsupported by evidence.

SGLT2 inhibitors have undoubtedly transformed CKD management in hyperfiltration-driven conditions. However, addressing the critical knowledge gap in their efficacy across diverse CKD subtypes and populations is essential to ensure evidence-based, personalized treatment strategies for all CKD patients.
